# Antimicrobial resistance among canine enterococci in the northeastern United States, 2007–2020

**DOI:** 10.3389/fmicb.2022.1025242

**Published:** 2023-01-05

**Authors:** Marwan Osman, Craig Altier, Casey Cazer

**Affiliations:** ^1^Cornell Atkinson Center for Sustainability, Cornell University, Ithaca, NY, United States; ^2^Department of Public and Ecosystem Health, College of Veterinary Medicine, Cornell University, Ithaca, NY, United States; ^3^Department of Population Medicine and Diagnostic Sciences, College of Veterinary Medicine, Cornell University, Ithaca, NY, United States

**Keywords:** *Enterococcus* spp., antimicrobial resistance, epidemiology, canine, surveillance, temporal trends

## Abstract

**Introduction:**

Antimicrobial resistance (AMR) is a growing and complex One Health concern worldwide, threatening the practice of human and veterinary medicine. Although dogs are a potential reservoir of multidrug-resistant bacteria, there are very few surveillance studies on AMR from the canine population in the United States. Here, we assessed the antimicrobial susceptibility patterns, identified temporal resistance and minimum inhibitory concentration trends, and described associations between resistance phenotypes among canine clinical enterococci in the northeastern United States.

**Methods:**

Through a large-scale retrospective study design, we collected species identification, minimum inhibitory concentration, and clinical data from 3,659 canine enterococci isolated at the Cornell University Animal Health Diagnostic Center between 2007 and 2020. We used the Mann-Kendall test, Sen’s slope, multivariable logistic regression, and survival analysis models to detect the presence of a significant trend in resistance over the study period.

**Results:**

*Enterococcus faecalis* was the most prevalent species (67.1% of isolates), followed by *Enterococcus faecium* (20.4%). We found high levels of AMR among enterococci to almost all the tested antimicrobials, particularly *E. faecium*. The lowest percentage of resistance was to vancomycin and chloramphenicol. Multidrug resistance was common (80% of *E. faecium* and 33% of *E. faecalis*) and 31 isolates were extensively drug resistant. Multidrug resistance among *E. faecium* increased over time, but not in *E. faecalis*. Resistance to penicillins, enrofloxacin, and rifampin increased during the study period, but resistance to tetracyclines is on a downward trajectory compared to AMR data from the last decade. Emerging vancomycin-resistant *E. faecalis* (0.3%) and *E. faecium* (0.8%) infections in the canine population are of great concern to both human and animal health. One *E. faecium* isolate with acquired vancomycin resistance was identified in 2017 and four vancomycin-resistant enterococci isolates were identified in 2020.

**Conclusion:**

There is a crucial need to make rational prescribing decisions on the prudent use of antimicrobials and improve the quality of care for patients, especially when empirical antimicrobial treatment for enterococcal infection is common.

## 1. Introduction

Antimicrobial resistance (AMR) has become one of the leading global public health challenges facing humanity, posing a major threat to human and animal health around the globe (Murray et al., 2022). Although AMR is a complex issue with many contributing factors, excessive use of antimicrobials in humans and animals represents the most important driving force toward the selection of bacteria with acquired resistance and subsequently the emergence and dissemination of AMR determinants (Holmes et al., 2016).

During the last few decades, the number of companion animals (e.g., dogs, cats, horses) in the United States has substantially increased and a change in their social role has occurred; the pet dog population has been recently estimated at nearly 77 million in the country, with approximately 38% of households having a dog (Overgaauw et al., 2020). Pet-associated bacterial infections represent a relatively neglected area compared with food-producing animal infections. Household pets live in close contact with humans and pose a substantial risk for transmission of illnesses and drug-resistant pathogens to susceptible owners, pet shop employees, veterinarians, as well as other animals (Rees et al., 2021). Novel resistance determinants continue to emerge in zoonotic pathogens and commensal bacteria isolated from household pets, mostly dogs and cats (Jackson et al., 2009; Leonard et al., 2011; Cummings et al., 2015; KuKanich and Lubbers, 2015; Zhang et al., 2016; Bourély et al., 2019; Li et al., 2021; Hata et al., 2022; Tóth et al., 2022). Dogs are increasingly recognized as a potential reservoir and a relevant transmission pathway of commensal and pathogenic bacteria or their resistance genes (Harada et al., 2012; Damborg et al., 2016; Francois Watkins et al., 2021).

Narrow- and broad-spectrum antimicrobial agents are used widely in veterinary medicine for therapeutic and prophylactic purposes in companion animals. Many of the antimicrobials are the same as or similar to those used in human medicine (Joosten et al., 2020). Prescriptions for antimicrobials important in human medicine to companion animals in the United States do not have to be reported, though they are common among veterinarians and legal (Papich, 2021). A direct relationship exists between excessive use of antimicrobials and the spread of drug-resistant bacteria, increasing the risk of antimicrobial treatment failure in both animals and humans (Llor and Bjerrum, 2014).

Among the animal commensal flora, enterococcal species have been commonly considered as a potential source of infections and resistance genes among humans. *Enterococcus* spp. are Gram-positive, catalase-negative, facultative anaerobic commensal bacteria that exist in chains or pairs and do not form spores, with the ability to grow in 6.5% NaCl broth and a particular resistance to drying and bile (Švec and Devriese, 2015). These natural inhabitants of the gastrointestinal tract and oral environment of mammals can cause opportunistic infections in humans and dogs and constitute a frequent reason for antimicrobial prescription (Komiyama et al., 2016; Ramos et al., 2020). Enterococcal species are a common cause of urinary tract and skin and soft tissue infections but also a major pathogen of concern responsible for life-threatening infections such as endocarditis, abscesses, meningitis, and bacteremia (Mercuro et al., 2018).

*Enterococcus* spp. are known to be intrinsically resistant to a number of antimicrobial agents, including cephalosporins, clindamycin, and colistin, and exhibit low-level resistance to β-lactams and aminoglycosides (Zaheer et al., 2020). The minimal inhibitory concentration (MIC) of enterococci to gentamicin typically ranges from 6 mg/l to as high as 48 mg/l. The facultative anaerobic metabolism of enterococci is most likely the reason of their intrinsic resistance to all aminoglycosides by reducing the transmembrane potential and thereby limiting drug uptake into the cell (Chow, 2000). The use of trimethoprim-sulfamethoxazole against enterococci is not appropriate and associated with adverse effects. Although enterococci appear sensitive *in vitro*, the antimicrobial is not effective *in vivo* and not recommended clinically (Wisell et al., 2008; Sykes, 2014). Additionally, enterococci are remarkable in their ability to survive their hosts (Tyne et al., 2019), acquire AMR determinants, and horizontally transfer antimicrobial-resistant determinants *via* genetic mobile elements to other enterococcal strains or different species such as *Staphylococcus aureus* and *Listeria monocytogenes* (Leclercq et al., 1989; Johnson and Woodford, 2002; González-Zorn and Courvalin, 2003; de Niederhäusern et al., 2004; Ahmed and Baptiste, 2018). *Enterococcus faecalis* and *Enterococcus faecium*, the most prevalent enterococci species encountered in human and animal infections, have become of increasing importance over recent decades (Barlow et al., 2017). Dogs have been described as potential reservoirs of drug-resistant enterococci in animals worldwide, but available data on resistant enterococci remain scarce in the United States. We aim to assess the antimicrobial susceptibility patterns, identify trends in resistance, and describe associations between resistance phenotypes among canine clinical enterococci isolates in the northeastern United States. Understanding the prevalence and temporal trends of AMR among dogs is critical to understand the One Health risk associated with antimicrobial use and AMR in companion animals.

## 2. Materials and methods

### 2.1. Study design, data source, and management

Retrospective clinical and minimum inhibitory concentration (MIC) records from *Enterococcus* spp. isolated from canines between July 19, 2007, and December 31, 2020 were analyzed in the present study. The data were provided by the Cornell University Animal Health Diagnostic Center (AHDC) in Ithaca, New York. The records were analyzed using R software (R Core team, version 4.1.0; R Studio, version 1.4.1106). The database was imported for cleaning, variable coding, and analysis. Descriptive analysis, models, and illustrations were done on all variables using several R packages (e.g., stringr, summarytools, prettyR, ggplot2, hrbrthemes, stats, Kendall, survival, icenReg). All code necessary to replicate the analysis is publicly available (DOI: 10.5281/zenodo.7126369).

The database was assessed for duplicates and missing information. According to the Clinical and Laboratory Standards Institute (CLSI) guideline regarding cumulative antibiograms reports, only one *Enterococcus* isolate per culture (our dataset lacked unique patient identifiers) was included in our investigation, regardless of the body site and antimicrobial susceptibility pattern. Subsequent isolates were identified and removed from the database. Variables collected from the laboratory information system included the species identification, date of the isolation, origin of clinical sample (body site), and MIC value for each antimicrobial agent. All enterococcal isolates were recovered from patients with clinically significant infections, including urinary tract, skin and soft tissues, reproductive system, and invasive infections.

### 2.2. Microbiological analysis and antimicrobial susceptibility testing

Microbiological identification at species level was performed using either the Sensititre Automated Microbiology System (TREK Diagnostic Systems, Cleveland, Ohio, USA) or Matrix-Assisted Laser Desorption/Ionization Time-Of-Flight mass spectrometry (MALDI Biotyper; Bruker, Bellerica, MA, USA). All procedures at the Cornell University AHDC were performed in accordance with accreditation by the American Association of Veterinary Laboratory Diagnosticians (AAVLD). Antimicrobial susceptibility testing of *Enterococcus* isolates was carried out using the broth microdilution method as previously described (Cummings et al., 2015). The Sensititre™ Gram Positive MIC Plates, panel CMV1BURF and COMPGP1F, were used for canine urinary and non-urinary *Enterococcus* spp. isolates, respectively. Quality control was performed weekly using *E. coli* ATCC 25922, *S. aureus* 29213, *E. faecalis* 29212, and *Pseudomonas aeruginosa* 27853. The MIC ranges for quality control recommended by the CLSI were used, and results were accepted if the MIC values were within expected ranges for these bacterial strains.

The MIC values were interpreted according to the interpretive criteria (i.e., breakpoints) recommended by the CLSI guidelines (CLSI-VET01S ED5: 2020; Human breakpoints were used as there are no animal enterococci breakpoints; Weinstein and Lewis, 2020) and, if a CLSI breakpoint was not available, veterinary antibiogram committee of the French Society for Microbiology (CA-SFM; www.sfm-microbiologie.fr) to classify isolates as susceptible or non-susceptible to each agent. The drugs selected for this study (Table 1) have pharmacologic activity against *Enterococcus* spp. and are clinically relevant to canine medicine, either through therapeutic use or as markers for susceptibility to commonly used antimicrobial agents. No clinical breakpoints are available in the CLSI/CA-SFM guidelines for enrofloxacin; thus, we adopted those from the veterinary CA-SFM guidelines for *Streptococcus*. Regardless of isolation year, all MIC values were interpreted using the same set of current guidelines. We excluded the rare cases of historical MIC values that could not be interpreted with the current CLSI or CA-SFM clinical breakpoints. The few isolates with intermediate susceptibility were categorized as being non-susceptible.

**Table 1:** Prevalence of antimicrobial resistance among *Enterococcus* spp. clinical isolates from dogs stratified by species, from canine clinical infections at the Cornell University Animal Health Diagnostic Center (AHDC), 2007–2020.

Antimicrobial category^b^	Antimicrobial agent	All enterococci		*Enterococcus faecalis*		*Enterococcus faecium*		*Enterococcus avium*		*Enterococcus canintestini*		*Enterococcus durans*		*Enterococcus casseliflavus*		*Enterococcus gallinarum*		*Enterococcus hirae*		Other *Enterococcus* spp. (*T* = 193)	
		(*T* = 3,659)		(*T* = 2,454)		(*T* = 748)		(*T* = 68)		(*T* = 61)		(*T* = 40)		(*T* = 38)		(*T* = 29)		(*T* = 28)			
		*N*	%*R*	*N*	%*R*	*N*	%*R*	*N*	%*R*	*N*	%*R*	*N*	%*R*	*N*	%*R*	*N*	%*R*	*N*	%*R*	*N*	%*R*
Penicillins (PEN)	Ampicillin	3436	13	2373	0.7	639	63.2	65	6.2	59	1.7	38	15.8	34	2.9	28	0	28	0	172	7
	Penicillin G	2327	16.5	1560	1	505	68.5	48	2.1	23	4.3	24	16.7	26	0	17	0	14	0	110	15.5
Glycopeptides (GLY)	Vancomycin	967^a^	7.4	592	0.3	249	0.8	16	0	19	5.3	1	0	38^a^	100	29^a^	100	10	0	13	0
Aminoglycosides (AMG)	Gentamicin^c^	936	-	621	-	201	-	14	-	23	-	9	-	12	-	8	-	9	-	39	-
Tetracyclines (TET)	Tetracycline	2013	31.1	1421	25.8	355	52.1	33	45.5	35	14.3	19	42.1	20	10	19	26.3	16	62.5	95	69.5
	Doxycycline	1689	25.6	1155	22.8	317	39.1	39	30.8	21	0	17	23.5	28	0	15	26.7	8	37.5	89	75.3
Macrolides (ERY)	Erythromycin	2357	72.7	1578	71.8	510	91	50	16	24	62.5	24	37.5	30	83.3	17	23.5	14	7.1	110	49.1
Phenicols (CHL)	Chloramphenicol	2358	7.3	1576	6.5	512	11.5	50	2	25	4	24	8.3	30	3.3	17	11.8	15	0	109	2.8
Fluoroquinolones (FQ)	Enrofloxacin^d^	3571	73.3	2418	68.5	736	93.1	66	90.9	45	46.7	38	50	34	91.2	29	86.2	28	35.7	177	61.6
Ansamycins (RIF)	Rifampin	2325	68.1	1559	72.5	505	74.7	48	12.5	23	4.3	24	29.2	26	76.9	17	41.2	14	21.4	109	30.3
Nitrofurans (FUR)	Nitrofurantoin^e^	911	29.4	587	2.2	249	91.6	16	93.8	18	11.1	1	100	11	9.1	8	0	10	40	11	36.4

*T*: total number of isolates. *N*: number of tested enterococcal isolates. %*R*: percentage of resistance. Clinical breakpoints were adopted from those related to humans (Clinical and Laboratory Standards Institute (CLSI) VET01S ED5:2020; Weinstein and Lewis, 2020).

a *Enterococcus casseliflavus* and *Enterococcus gallinarum* have an intrinsic low-level vancomycin resistance.

b The antimicrobial categories were adopted from Magiorakos et al. (2012).

c The tested antimicrobial concentrations do not allow categorizing the isolates as susceptible or non-susceptible.

d No clinical breakpoints are available in the CLSI VET01S ED5:2020. Clinical breakpoints were adopted from the guidelines of the Veterinary Antibiogram Committee of the French Society for Microbiology (CA-SFM; www.sfm-microbiologie.fr) for *Streptococcus* spp.

e There are no available clinical breakpoints for non-urinary isolates. The clinical breakpoints for urinary isolates have been applied to non-urinary isolates.

Although 11 antimicrobials (penicillin G, ampicillin, vancomycin, gentamicin, tetracycline, doxycycline, erythromycin, chloramphenicol, enrofloxacin, rifampicin, and nitrofurantoin) were tested throughout the study period, only ampicillin (*n* = 3,589 isolates tested out of 3,659) and enrofloxacin (*n* = 3571 isolates tested out of 3,659) were used on almost all *Enterococcus* spp. isolates. Vancomycin and nitrofurantoin were only consistently used after 2017. The susceptibility of urinary isolates was systematically assessed using a narrow antimicrobial susceptibility testing panel (CMV1BURF Sensititre plate), including ampicillin, tetracycline, and enrofloxacin. In the case of non-urinary isolates, the antimicrobial susceptibility testing panel was extended to the full list of antimicrobials, except tetracycline which was rarely tested for non-urinary isolates. On the other hand, in few specific cases (e.g., multidrug-resistant (MDR) isolates) of *Enterococcus* urinary tract infections, the susceptibility was assessed using the larger non-urinary panel. We did not report the percentage of resistance against these antimicrobials among urinary isolates when fewer than 5% of the isolates representing a species were tested (Table 2). Given that *Enterococcus gallinarum* and *Enterococcus casseliflavus* have intrinsic low-level vancomycin resistance (Monticelli et al., 2018), we categorized the respective isolates as resistant to vancomycin regardless of their MIC values.

**Table 2:** Prevalence of antimicrobial resistance among *Enterococcus* spp. clinical isolates from dogs stratified by sample source, from canine clinical infections at the Cornell University Animal Health Diagnostic Center (AHDC), 2007–2020.

Antimicrobial agent	Resistance rate																							
	*Enterococcus faecalis*												*Enterococcus faecium*											
	Urinary (*T* = 891)		Skin and soft tissues (*T* = 1,038)		Reproductive system (*T* = 203)		Invasive (*T* = 104)		Intestinal (*T* = 98)		Unspecified location (*T* = 120)		Urinary (*T* = 281)		Skin and soft tissues (*T* = 142)		Reproductive system (*T* = 38)		Invasive (*T* = 122)		Intestinal (*T* = 145)		Unspecified location (*T* = 20)	
	*N*	%*R*	*N*	%*R*	*N*	%*R*	*N*	%*R*	*N*	%*R*	*N*	%*R*	*N*	%*R*	*N*	%*R*	*N*	%*R*	*N*	%*R*	*N*	%*R*	*N*	%*R*
Ampicillin	878	1.1	981	0.4	198	0.5	100	1.0	96	1.0	120	0	262	67.9	99	48.5	33	30.3	90	61.1	140	75.0	15	53.3
Penicillin G	35	NS^a^	1012	1.1	200	1.0	101	1.0	98	1.0	114	0	54	75.9	131	62.6	38	39.5	119	71.1	144	77.1	19	63.2
Vancomycin	7	NS^b^	390	0.3	83	0	27	0	53	0	32	0	22	0	47	4.3	11	0	37	0	127	0	5	0
Tetracycline	859	35.6	366	9.0	73	13.7	43	16.3	36	5.6	44	20.5	246	62.6	30	46.7	7	57.1	26	34.6	40	5.0	6	33.3
Doxycycline	23	NS	755	21.5	151	25.2	73	31.5	70	15.7	83	26.5	39	48.7	94	37.2	25	36.0	88	53.4	58	13.8	13	46.2
Erythromycin	35	NS	1031	73.1	200	71.0	101	72.3	97	61.9	114	73.7	54	96.3	136	91.2	38	76.3	119	85.7	144	97.2	19	89.5
Chloramphenicol	35	NS	1027	5.9	201	7.5	101	2.0	98	9.2	114	11.4	54	5.6	137	10.2	38	5.3	119	16.0	145	12.4	19	15.8
Enrofloxacin^c^	881	65.5	1014	70.0	201	68.2	104	68.3	98	73.5	120	75.0	279	91.8	132	95.5	38	71.1	122	93.4	145	98.6	20	95.0
Rifampin	35	NS	1011	72.4	200	75.5	101	67.3	98	76.5	114	67.5	54	75.9	131	67.9	38	50.0	119	78.2	144	85.4	19	63.2
Nitrofurantoin	7	NS	387	2.1	82	3.7	27	3.7	53	1.9	31	0	22	90.9	47	95.7	11	100	37	97.3	127	88.2	5	80.0

*T:* total number of isolates. *N*: number of tested enterococcal isolates. %R: percentage of resistance. Clinical breakpoints were adopted from those related to humans (Clinical and Laboratory Standards Institute (CLSI) VET01S ED5:2020; Weinstein and Lewis, 2020).

a NS: Resistance data is not shown because less than 5% of the total number of isolates were tested against the antimicrobial; thus, the available information does not reflect the true non-susceptibility rate.

b Only seven *Enterococcus faecalis* isolates were tested for vancomycin; one isolate was vancomycin-resistant.

c No clinical breakpoints are available in the CLSI VET01S ED5:2020. Clinical breakpoints were adopted from the guidelines of the Veterinary Antibiogram Committee of the French Society for Microbiology (CA-SFM; www.sfm-microbiologie.fr) for *Streptococcus* spp.

### 2.3. Definition of multidrug resistant isolates

We divided our isolates into two main groups, *E. faecalis* and *E. faecium* isolates. MDR isolates were defined as acquired non-susceptibility to at least one agent in three or more antimicrobial categories (Magiorakos et al., 2012). Extremely drug resistant (XDR) isolates were defined as *in vitro* acquired non-susceptibility to at least one antimicrobial drug in all but two or fewer antimicrobial categories (Magiorakos et al., 2012). We defined nine categories: penicillins (PEN; penicillin G and ampicillin), glycopeptides (GLY; vancomycin), tetracyclines (TET; tetracycline and doxycycline), macrolides (ERY; erythromycin), phenicols (CHL; chloramphenicol), fluoroquinolones (FQ; enrofloxacin), ansamycins (RIF; rifampin), and nitrofurans (FUR; nitrofurantoin; Table 1). The tested MIC values for gentamicin did not allow us to interpret isolates as susceptible or resistant with the current breakpoint; thus, we excluded the aminoglycoside category in our MDR definition.

### 2.4. Statistical analysis

Descriptive and statistical analysis were performed using the R software. The mean, standard deviation, and range of *Enterococcus* isolates per year was calculated. The categorical data was presented as frequencies and associated proportions. For each antimicrobial agent, the differences in resistance trends across *E. faecalis* and *E. faecium* were initially compared using the chi-squared test. The Mann–Kendall test (MKT) and Sen’s slope were used to detect temporal trends of antimicrobial monoresistance and multidrug resistance among *E. faecalis* and *E. faecium* isolates over the study period (2007–2020). Subsequently, using multivariable logistic regression (MLR), we modeled resistance to antimicrobials for *E. faecalis* and *E. faecium* accounting for both body site and time, divided into four periods: (1) 2007–2010, (2) 2011–2014, (3) 2015–2017, and (4) 2018–2020. Resistance to the antimicrobial was the outcome and body site and study period were the explanatory variables. We analyzed MIC distributions with Cox proportional hazards regression models for all 12 tested antimicrobials. Briefly, the inhibition of bacterial growth was considered as the event; thus, we analyzed the concentration of antimicrobial required to achieve the event (i.e., MIC), instead of time to event. In this context, resistance trends can be analyzed over an entire range of concentrations and no specific breakpoint value for resistance has to be determined. A separate model was created for each tested antimicrobial with species identification, body site, and study period as the explanatory variables. A Hazard Ratio (HR) has been calculated indicating a higher (HR > 1) or lower (HR < 1) likelihood of growth inhibition of the studied *Enterococcus* group at each antimicrobial concentration compared to a reference *Enterococcus* group (Spruance et al., 2004; Combescure et al., 2014; Osman et al., 2022). We assessed the assumption of proportional hazards visually by examining the survival curves. MLR models were also used to predict resistance to each of the regularly used antimicrobials with co-resistant and cross-resistant agents among *Enterococcus* spp. and the *E. faecalis* and *E. faecium* subpopulations. Antimicrobials within the same category were removed from the statistical models (e.g., penicillin was excluded from models to predict ampicillin resistance). All statistical tests were two-sided, with a type I error set at *α* = 0.05. Backward stepwise model selection was used to better identify the associations of covariates with the outcome antimicrobial in MLR models. To decrease the false discovery rate in our statistical analyses, we performed the Benjamini-Hochberg method to adjust the calculated *p*-values in each table, with a false discovery rate of 0.05 (Benjamini and Hochberg, 1995).

## 3. Results

A total of 3,659 canine *Enterococcus* spp. unique isolates (one isolate per culture) were collected at the Cornell University AHDC during a 14-year period (2007–2020). These isolates were mostly obtained from urine (*N* = 1,344; 36.7%), followed by skin and soft tissues (N = 1,324; 36.2%), reproductive system (*N* = 319; 8.7%), invasive locations (*N* = 261; 7.1%), intestinal tract (*N* = 252; 6.9%), and other locations (*N* = 159; 4.3%). Eleven different *Enterococcus* spp. were isolated from canine clinical specimens. The predominant species identified was *E. faecalis* (*N* = 2,454; 67.1%), followed by *E. faecium* (*N* = 748; 20.4%), *Enterococcus avium* (*N* = 68; 1.9%), *Enterococcus canintestini* (*N* = 61; 1.7%), *Enterococcus durans* (*N* = 40; 1.1%), *E. casseliflavus* (*N* = 38; 1.0%), *E. gallinarum* (*N* = 29; 0.8%), *Enterococcus hirae* (*N* = 28; 0.8%), *Enterococcus canis* (*N* = 5; 0.1%), *Enterococcus raffinosus* (*N* = 5; 0.1%), and *Enterococcus mundtii* (*N* = 1; 0.0%). The remaining isolates (*N* = 182, 5.0%) were not identified at species level. Overall, the mean number of *Enterococcus* spp. isolated per year was 261 (standard deviation [SD]: 77, range: 87–369), with 175 (SD: 49, range: 67–248) *E. faecalis* isolates and 53 (SD: 24, range: 11–98) *E. faecium* isolates per year.

*Enterococcus faecalis* isolates were mainly obtained from skin and soft tissues (*N* = 1038, 42.3%) and urine (*N* = 891, 36.3%). However, *E. faecium* was isolated from broader specimen types including urine (*N* = 281, 37.6%), intestinal tract (*N* = 145; 19.4%), skin and soft tissues (*N* = 142, 19.0%), and invasive locations (*N* = 122, 16.3%; Table 2).

The prevalence of resistance to each antimicrobial across the study period, stratified by species, is summarized in Table 1. Antimicrobial susceptibility testing showed a relatively low resistance rate to chloramphenicol (7.3% resistant), vancomycin (7.4%), and penicillins (13%–16.5%) among *Enterococcus* spp. isolates. Higher percentages of resistance were observed against tetracyclines (25.6% resistant to doxycycline and 31.1% to tetracycline), nitrofurantoin (29.4%), rifampin (68.1%), erythromycin (72.7%), and enrofloxacin (73.3%).

Of note, only three antimicrobials were consistently tested on *E. faecalis* and *E. faecium* urinary isolates: ampicillin, tetracycline, and enrofloxacin (Table 2). After dividing the study years into four periods and accounting for year of isolation, MLR analysis demonstrated that *Enterococcus* spp. non-urinary isolates were significantly less likely than urinary isolates to present *in vitro* resistance to tetracycline (odds ratio (OR) = 0.10–0.39; *p* < 0.05; Table 3). However, intestinal *Enterococcus* isolates showed the highest rates of resistance to ampicillin (OR = 3.51; 95% confidence interval (95% CI) = 2.58 to 4.77; *p* < 0.001) and enrofloxacin (OR = 2.70; 95% CI = 1.84 to 4.06; *p* < 0.001) compared to urinary isolates. In addition, invasive isolates were more likely to be resistant to the abovementioned antimicrobials (*p* < 0.01) compared to urinary isolates (Table 3). In contrast, after accounting for species, body site, and study period, we only found higher MICs against enrofloxacin among intestinal and invasive isolates compared to urinary isolates (Supplementary Table S1). Compared to *E. faecalis*, isolates from *E. faecium* were more resistant to all the tested antimicrobials, particularly penicillins (63.2% ampicillin resistant and 68.5% penicillin resistant in *E. faecium* versus 0.75 and 1.0% in *E. faecalis*, *p* ≤ 0.001), vancomycin (0.8% versus 0.3%, *p* = 0.729), tetracyclines (39.1% doxycycline resistant and 52.1% tetracycline resistant versus 22.8and 25.8%, *p* ≤ 0.001), erythromycin (91.0% versus 71.8%, *p* ≤ 0.001), enrofloxacin (93.1% versus 68.5%, *p* ≤ 0.001), and nitrofurantoin (91.6% versus 2.2%, *p* ≤ 0.001). Survival analysis models concurred with changes in the percent of resistant isolates. *Enterococcus* other than *faecalis* and *faecium* showed a decrease in MIC values for penicillin (*p* ≤ 0.001), gentamicin (*p* ≤ 0.001), erythromycin (i ≤ 0.001), chloramphenicol (*p* ≤ 0.001), and rifampin (*p* ≤ 0.001) but an increase in MIC values for enrofloxacin (*p* ≤ 0.001) compared to the reference *E. faecalis* (Supplementary Table S1).

**Table 3:** Determinants of resistance to the common antimicrobials including specimen source and study period among *Enterococcus* spp. isolates using multivariable logistic regression models, in canine clinical infections at the Cornell University Animal Health Diagnostic Center (AHDC), 2007–2020.

	*Enterococcus* spp.				*Enterococcus faecalis*				*Enterococcus faecium*			
	Model^a^				Model^a^				Model^a^			
	Adj. OR	95% CI	*p*-value	Adj. *p*-value^c^	Adj. OR	95% CI	*p*-value	Adj. *p*-value^c^	Adj. OR	95% CI	*p*-value	Adj. *p*-value^c^
Resistance to ampicillin												
Urinary tract^b^												
Intestinal	**3.51**	**2.58–4.77**	**<0.001**	**<0.001**	0.76	0.04–4.10	0.792	0.859	0.76	0.45–1.28	0.291	0.458
Invasive	**1.98**	**1.40–2.77**	**<0.001**	**0.001**	0.93	0.05–4.97	0.944	0.987	0.57	0.34–0.98	0.042	0.114
Unspecified site	**0.32**	**0.14–0.62**	**0.002**	**0.009**	28*10^−6^	0–24*10^20^	0.988	0.994	0.45	0.15–1.41	0.159	0.321
Reproductive system	**0.30**	**0.17–0.49**	**<0.001**	**<0.001**	0.39	0.02–2.10	0.379	0.519	**0.18**	**0.08–0.41**	**<0.001**	**<0.001**
Skin and soft tissues	**0.25**	**0.19–0.34**	**<0.001**	**<0.001**	0.32	0.09–0.98	0.058	0.141	**0.31**	**0.19–0.52**	**<0.001**	**<0.001**
Isolation date (2007–2010)^b^												
Isolation date (2011–2014)	**0.33**	**0.23–0.48**	**<0.001**	**<0.001**	0.49	0.06–2.95	0.431	0.571	**0.31**	**0.18–0.53**	**<0.001**	**<0.001**
Isolation date (2015–2017)	0.72	0.53–0.99	0.044	0.115	1.29	0.31–6.32	0.733	0.813	1.30	0.76–2.22	0.335	0.494
Isolation date (2018–2020)	1.42	1.07–1.90	0.015	0.052	1.91	0.52–9.00	0.354	0.503	**2.18**	**1.34–3.54**	**0.002**	**0.007**
Resistance to penicillin G												
Urinary tract^b^												
Intestinal	0.88	0.55–1.43	0.616	0.735	0.32	0.01–8.35	0.428	0.570	0.70	0.31–1.50	0.369	0.513
Invasive	0.78	0.49–1.27	0.319	0.485	0.33	0.01–8.62	0.443	0.580	0.87	0.39–1.84	0.717	0.809
Unspecified site	**0.16**	**0.08–0.30**	**<0.001**	**<0.001**	10^−6^	0–48*10^21^	0.987	0.994	0.50	0.16–1.65	0.246	0.411
Reproductive system	**0.10**	**0.05–0.18**	**<0.001**	**<0.001**	0.32	0.03–7.05	0.360	0.508	**0.22**	**0.08–0.54**	**0.001**	**0.006**
Skin and soft tissues	**0.10**	**0.07–0.16**	**<0.001**	**<0.001**	0.35	0.06–6.47	0.321	0.485	0.51	0.24–1.06	0.079	0.182
Isolation date (2007–2010)^b^												
Isolation date (2011–2014)	0.72	0.48–1.08	0.114	0.246	1.48	0.28–10.7	0.655	0.763	0.83	0.45–1.49	0.525	0.650
Isolation date (2015–2017)	1.32	0.90–1.93	0.154	0.314	1.77	0.38–12.5	0.499	0.626	**2.17**	**1.19–4.02**	**0.012**	**0.044**
Isolation date (2018–2020)	**1.62**	**1.13–2.33**	**0.009**	**0.033**	1.61	0.34–11.4	0.576	0.700	**2.86**	**1.57–5.26**	**<0.001**	**0.004**
Resistance to tetracycline												
Urinary tract^b^												
Intestinal	**0.10**	**0.03–0.25**	**<0.001**	**<0.001**	**0.13**	**0.02–0.43**	**0.005**	**0.020**	**0.06**	**0.01–0.20**	**<0.001**	**0.001**
Invasive	**0.34**	**0.19–0.58**	**<0.001**	**0.001**	**0.29**	**0.12–0.65**	**0.004**	**0.018**	0.35	0.13–0.89	0.030	0.088
Unspecified site	**0.39**	**0.20–0.74**	**0.005**	**0.021**	0.43	0.19–0.89	0.031	0.089	0.21	0.03–1.19	0.088	0.201
Reproductive system	**0.28**	**0.16–0.47**	**<0.001**	**<0.001**	**0.32**	**0.15–0.61**	**0.001**	**0.006**	0.69	0.13–3.99	0.656	0.763
Skin and soft tissues	**0.19**	**0.13–0.26**	**<0.001**	**<0.001**	**0.18**	**0.12–0.27**	**<0.001**	**<0.001**	0.60	0.26–1.41	0.242	0.411
Isolation date (2007–2010)^b^												
Isolation date (2011–2014)	**0.60**	**0.44–0.81**	**<0.001**	**0.004**	**0.58**	**0.40–0.83**	**0.003**	**0.013**	0.95	0.44–2.03	0.886	0.945
Isolation date (2015–2017)	**0.50**	**0.37–0.66**	**<0.001**	**<0.001**	**0.58**	**0.41–0.82**	**0.002**	**0.009**	0.64	0.30–1.37	0.251	0.415
Isolation date (2018–2020)	**0.33**	**0.25–0.44**	**<0.001**	**<0.001**	**0.37**	**0.26–0.52**	**<0.001**	**<0.001**	**0.19**	**0.10–0.36**	**<0.001**	**<0.001**
Resistance to doxycycline												
Urinary tract^b^												
Intestinal	**0.38**	**0.18–0.77**	**0.008**	**0.031**	0.54	0.17–1.76	0.296	0.461	0.28	0.09–0.86	0.030	0.088
Invasive	0.99	0.52–1.82	0.968	0.994	1.11	0.39–3.38	0.847	0.908	0.76	0.30–1.86	0.549	0.672
Unspecified site	0.62	0.30–1.18	0.167	0.330	0.85	0.30–2.57	0.767	0.841	0.52	0.13–2.07	0.352	0.503
Reproductive system	0.51	0.27–0.93	0.032	0.091	0.98	0.36–2.84	0.976	0.994	0.30	0.09–0.92	0.039	0.108
Skin and soft tissues	**0.44**	**0.25–0.76**	**0.003**	**0.013**	0.70	0.28–1.92	0.470	0.602	0.41	0.16–0.98	0.049	0.124
Isolation date (2007–2010)^b^												
Isolation date (2011–2014)	0.79	0.55–1.15	0.215	0.397	1.09	0.67–1.82	0.725	0.809	0.64	0.30–1.32	0.229	0.403
Isolation date (2015–2017)	0.77	0.53–1.12	0.171	0.331	1.03	0.63–1.73	0.906	0.956	0.62	0.28–1.34	0.229	0.403
Isolation date (2018–2020)	**0.01**	**0.00–0.03**	**<0.001**	**<0.001**	21*10^−7^	ND^d^	0.962	0.994	**0.02**	**0–0.07**	**<0.001**	**<0.001**
Resistance to erythromycin												
Urinary tract^b^												
Intestinal	1.38	0.76–2.43	0.274	0.440	1.46	0.65–3.23	0.353	0.503	0.72	0.09–4.01	0.722	0.809
Invasive	0.83	0.47–1.42	0.498	0.626	1.99	0.88–4.45	0.095	0.216	0.25	0.04–0.95	0.076	0.178
Unspecified site	0.73	0.40–1.32	0.305	0.469	2.22	0.99–4.93	0.049	0.124	0.29	0.03–2.66	0.243	0.411
Reproductive system	0.55	0.32–0.92	0.027	0.080	2.09	0.98–4.38	0.053	0.131	0.14	0.02–0.60	0.017	0.056
Skin and soft tissues	0.74	0.44–1.18	0.220	0.403	2.30	1.13–4.58	0.018	0.061	0.39	0.06–1.51	0.229	0.403
Isolation date (2007–2010)^b^												
Isolation date (2011–2014)	0.87	0.65–1.16	0.347	0.503	1.00	0.69–1.43	0.994	0.994	0.84	0.38–1.80	0.649	0.763
Isolation date (2015–2017)	0.79	0.59–1.05	0.110	0.239	0.66	0.46–0.93	0.019	0.061	3.37	1.27–10.1	0.020	0.063
Isolation date (2018–2020)	0.75	0.56–0.99	0.043	0.114	**0.53**	**0.38–0.75**	**<0.001**	**0.002**	**4.28**	**1.58–13.1**	**0.006**	**0.023**
Resistance to chloramphenicol												
Urinary tract^b^												
Intestinal	1.91	0.81–5.29	0.169	0.330	1.22	0.34–5.80	0.776	0.847	2.11	0.66–0.39	0.254	0.417
Invasive	1.63	0.68–4.55	0.303	0.469	0.23	0.03–1.47	0.119	0.253	3.55	1.13–15.8	0.052	0.129
Unspecified site	1.88	0.74–5.40	0.207	0.386	1.50	0.45–6.85	0.550	0.672	2.85	0.48–17.0	0.232	0.404
Reproductive system	1.16	0.48–3.24	0.756	0.835	0.96	0.29–4.31	0.946	0.987	1.02	0.13–6.55	0.981	0.994
Skin and soft tissues	1.00	0.46–2.64	0.994	0.994	0.72	0.25–3.08	0.601	0.721	1.92	0.59–8.64	0.323	0.485
Isolation date (2007–2010)^b^												
Isolation date (2011–2014)	0.82	0.50–1.37	0.449	0.583	0.75	0.41–1.38	0.352	0.503	1.07	0.36–3.20	0.904	0.956
Isolation date (2015–2017)	1.47	0.94–2.36	0.099	0.221	1.13	0.65–2.00	0.680	0.786	2.71	1.12–7.34	0.035	0.098
Isolation date (2018–2020)	0.81	0.49–1.33	0.387	0.523	0.57	0.31–1.06	0.075	0.178	1.60	0.64–4.40	0.331	0.492
Resistance to enrofloxacin												
Urinary tract^b^												
Intestinal	**2.70**	**1.84–4.06**	**<0.001**	**<0.001**	1.45	0.92–2.37	0.121	0.253	1.71	0.44–11.3	0.494	0.626
Invasive	**1.77**	**1.29–2.47**	**<0.001**	**0.003**	1.20	0.78–1.88	0.421	0.565	1.48	0.65–3.70	0.375	0.518
Unspecified site	1.60	1.09–2.42	0.021	0.065	1.64	1.07–2.58	0.027	0.080	1.49	0.26–28.2	0.712	0.809
Reproductive system	0.86	0.66–1.13	0.275	0.440	1.14	0.82–1.59	0.444	0.580	**0.21**	**0.09–0.53**	**<0.001**	**0.004**
Skin and soft tissues	1.13	0.95–1.34	0.164	0.329	1.22	1.01–1.49	0.044	0.115	1.76	0.72–4.96	0.244	0.411
Isolation date (2007–2010)^b^												
Isolation date (2011–2014)	1.18	0.95–1.46	0.135	0.279	1.26	0.98–1.61	0.071	0.171	1.33	0.70–2.56	0.385	0.523
Isolation date (2015–2017)	**1.90**	**1.51–2.38**	**<0.001**	**<0.001**	**1.86**	**1.43–2.41**	**<0.001**	**<0.001**	**11.3**	**3.71–49.7**	**<0.001**	**0.001**
Isolation date (2018–2020)	**1.43**	**1.15–1.77**	**0.001**	**0.007**	1.23	0.96–1.59	0.103	0.227	**45.3**	**8.94–829**	**<0.001**	**0.002**
Resistance to rifampin												
Urinary tract^b^												
Intestinal	1.33	0.78–2.27	0.290	0.458	0.83	0.31–2.03	0.688	0.786	1.18	0.51–2.67	0.688	0.786
Invasive	0.95	0.56–1.57	0.831	0.896	0.59	0.23–1.40	0.246	0.411	1.30	0.58–2.84	0.520	0.649
Unspecified site	0.70	0.40–1.21	0.204	0.385	0.58	0.22–1.36	0.227	0.403	0.46	0.14–1.54	0.197	0.379
Reproductive system	0.68	0.41–1.10	0.121	0.253	0.81	0.32–1.85	0.633	0.751	0.34	0.13–0.86	0.024	0.075
Skin and soft tissues	0.85	0.53–1.32	0.468	0.602	0.69	0.29–1.48	0.366	0.513	0.65	0.30–1.36	0.266	0.432
Isolation date (2007–2010)^b^												
Isolation date (2011–2014)	1.18	0.91–1.53	0.203	0.385	**1.55**	**1.12–2.13**	**0.008**	**0.030**	0.85	0.46–1.55	0.595	0.718
Isolation date (2015–2017)	**1.73**	**1.33–2.26**	**<0.001**	**<0.001**	**2.11**	**1.51–2.96**	**<0.001**	**<0.001**	**3.49**	**1.80–6.96**	**<0.001**	**0.002**
Isolation date (2018–2020)	**1.89**	**1.46–2.46**	**<0.001**	**<0.001**	**1.88**	**1.36–2.59**	**<0.001**	**0.001**	**2.95**	**1.57–5.60**	**<0.001**	**0.004**

a Origin of clinical sample and date of isolation (divided into four periods: 2007–2010, 2011–2014, 2015–2017, and 2018–2020) were entered in the model.

b Reference group.

c *p*-values were adjusted according to Benjamini and Hochberg (1995).

d ND: We are not able to determine the exact 95% confidence interval.

Bold values are significant results (*p*<0.05 after Benjamini and Hochberg adjustment).

**Figure 1:**
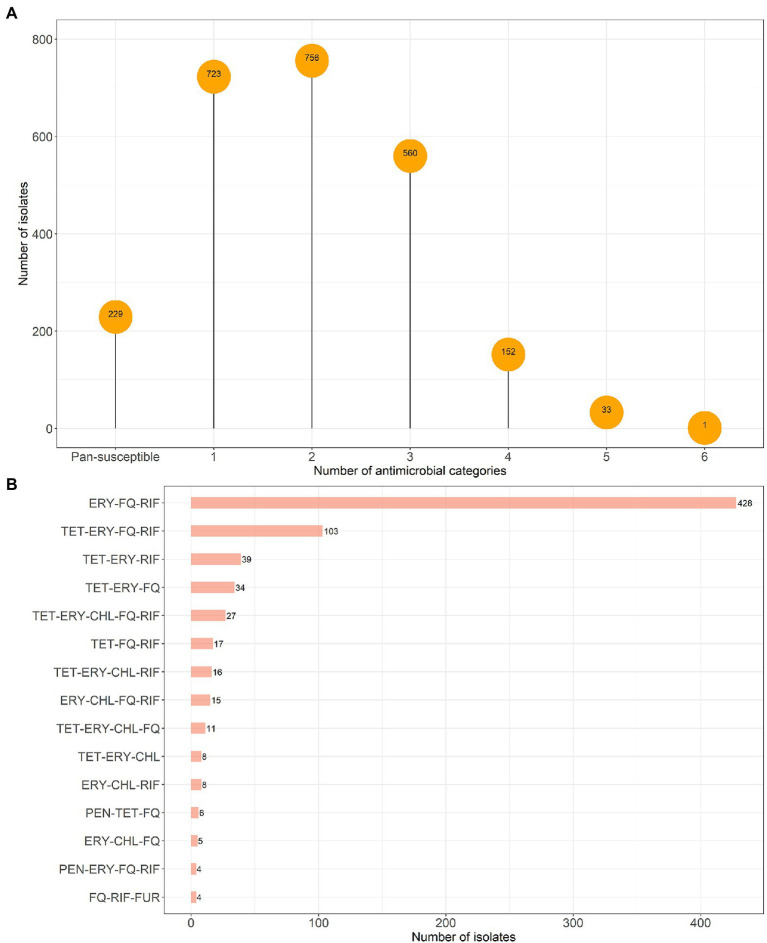
Distribution of resistance by number of antimicrobial categories **(A)** and most 15 common multidrug resistance patterns among *Enterococcus faecalis* isolates **(B)**, in canine clinical infections at the Cornell University Animal Health Diagnostic Center (AHDC), 2007–2020. Antimicrobial category abbreviations are listed in Table 1.

**Figure 2:**
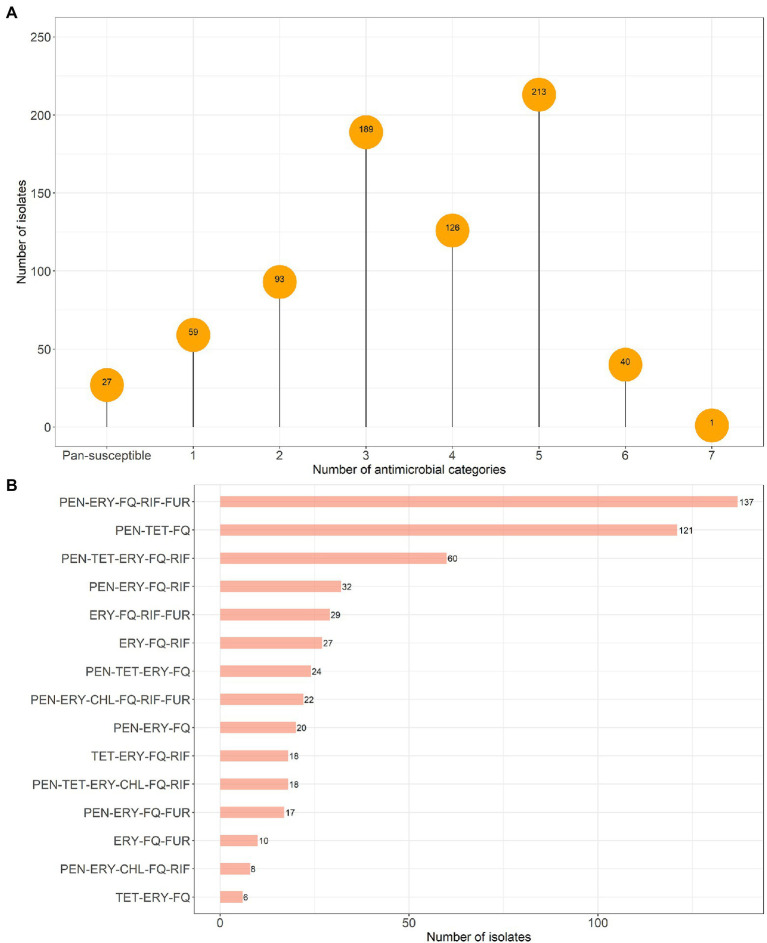
Distribution of resistance by number of antimicrobial categories **(A)** and most 15 common multidrug resistance patterns among *Enterococcus faecium* isolates **(B)**, in canine clinical infections at the Cornell University Animal Health Diagnostic Center (AHDC), 2007–2020. Antimicrobial category abbreviations are listed in Table 1.

Our data showed that the pan-susceptible pattern was uncommon among both *E. faecalis* (*N* = 229/2454, 9.3%; Figure 1A) and *E. faecium* (*N* = 27/748, 3.6%; Figure 2A). The rates of monoresistance (29.5%) and biresistance (30.8%) patterns were higher in *E. faecalis* compared to those in *E. faecium* (7.9% monoresistance, 12.4% biresistance). MDR, defined as *in vitro* acquired non-susceptibility to at least one drug in three or more antimicrobial categories (Magiorakos et al., 2012), was more frequently observed among *E. faecium* (76.1%) compared to *E. faecalis* (30.4%). Most MDR *E. faecium* isolates (82.6%) showed resistance to penicillins, but penicillin resistance was rare among MDR *E. faecalis* (2.9%). The most common multidrug resistance pattern among MDR *E. faecalis* isolates was erythromycin-fluoroquinolones-rifampin (57.4%, 428/746), followed by the same resistance pattern with an additional resistance to tetracycline (13.8%, 103/746; Figure 1B). *Enterococcus faecium* isolates were resistant to more antimicrobial classes (Figure 2B): penicillins-erythromycin-fluoroquinolones-rifampin-nitrofurantoin (24.1%, 137/569) was predominant, followed by penicillins-tetracycline-fluoroquinolones (21.3%, 121/569) and penicillins-tetracycline-erythromycin-fluoroquinolones-rifampin (10.5%, 60/569). XDR pattern, defined as *in vitro* acquired non-susceptibility to at least one antimicrobial drug in all but two or fewer antimicrobial categories (Magiorakos et al., 2012), was observed in *E. faecium* (*N* = 41) and *E. faecalis* (*N* = 1) isolates.

**Figure 3:**
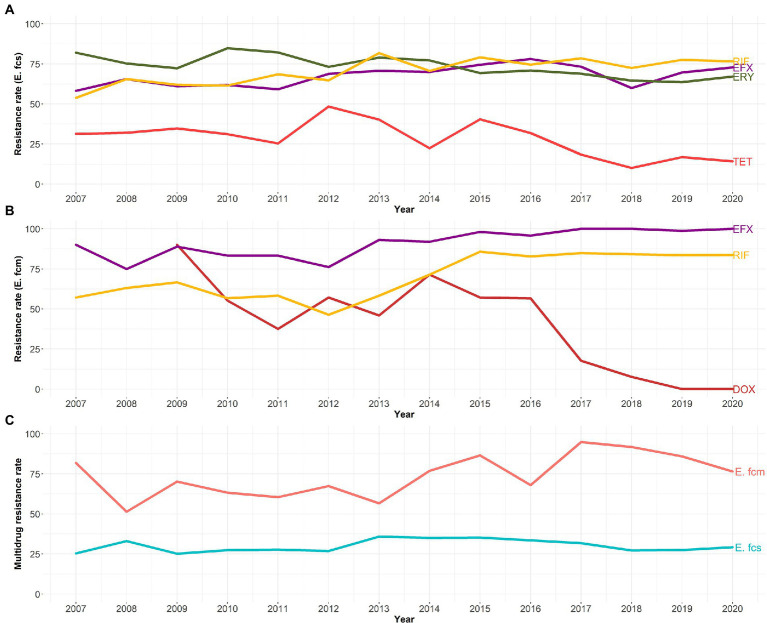
Temporal trends in the prevalence of resistance to tetracycline (TET), doxycycline (DOX), erythromycin, enrofloxacin (EFX), and/or rifampin (RIF; **A**, **B**), and multidrug resistance **(C)** among canine *Enterococcus faecalis* (*E. fcs*) and *Enterococcus faecium* (*E. fcm*) isolates during the study period (2007–2020), in canine clinical infections at the Cornell University Animal Health Diagnostic Center (AHDC), 2007–2020.

The proportion of *E. faecalis* that were MDR increased, generally, by 0.2 percentage points each year but the trend was not statistically significant (*Z* = 0.77, Sen’s = 0.002, *p*-value = 0.443). Similarly, *E. faecium* MDR proportion increased by 2 percentage points each year, but the trend was not statistically significant by the MKT (*Z* = 1.75, Sen’s = 0.021, *p*-value = 0.080). There was a statistically significant increase in the percent of isolates resistant to enrofloxacin [*E. faecalis*: increase of 1.1 percentage point per year (*Z* = 2.19, Sen’s = 1.064, *p*-value = 0.029), *E. faecium*: increase of 1.4 percentage points per year (*Z* = 3.20, Sen’s = 1.379, *p*-value = 0.001)] and rifampin [*E. faecalis*: increase of 1.5 percentage points per year (*Z* = 2.52, Sen’s = 1.510, *p*-value = 0.011), *E. faecium*: increase of 2.3 percentage points per year (*Z* = 2.47, Sen’s = 2.300, *p*-value = 0.014)]. However, the MKT and Sen’s slope showed a significant decreasing temporal resistance trend to tetracyclines among both *E. faecalis* (decrease of 1.5 percentage points per year for tetracycline, *Z* = −2.08, Sen’s = −1.487, *p*-value = 0.038) and *E. faecium* (decrease of 7.1% points per year for doxycycline, Z = −2.55, Sen’s = −7.109, *p*-value = 0.011). Moreover, erythromycin resistance is decreasing over time among *E. faecalis* isolates (decrease of 1.4 percentage points per year, *Z* = −3.18, Sen’s = −1.436, *p*-value = 0.001; Figure 3).

MLR analysis concurred with the MKT and Sen’s slope findings for both *E. faecalis* and *E. faecium*, identifying a decrease in resistance to tetracycline and increase in resistance to enrofloxacin and rifampin over time was observed after accounting for body site isolates (Table 3). Regarding *E. faecium*, MLR analysis has not only confirmed the MKT and Sen’s slope results, but also showed a significant increase in the level of resistance to ampicillin (OR = 2.18; 95% CI = 1.34 to 3.54; *p* = 0.007), penicillin (OR = 2.86; 95% CI = 1.57 to 5.26; *p* = 0.004), and erythromycin (OR = 4.28; 95% CI = 1.58 to 13.1; *p* = 0.023) among the circulating isolates in the 2018–2020 period compared to those isolated between 2007 and 2010. As for tetracycline, *E. faecium* isolates from 2018 to 2020 showed a lower resistance rate to doxycycline compared to peers isolated between 2007 and 2010 (OR = 0.02; 95% CI = 0.00 to 0.07; *p* < 0.001). Compared to the reference period (2007–2010), survival analysis models concurred with MLR findings among *E. faecium* isolates from 2018 to 2020. In addition, survival analysis confirmed MLR findings for rifampin, showing an increase in MIC values for this antimicrobial among *E. faecalis* isolated from 2018 to 2020 (HR = 0.69; CI = 0.57–0.84; *p* = 0.002), and also revealed an increase in MIC values for ampicillin (HR = 0.71; CI = 0.58–0.88; *p* = 0.011) and penicillin (HR = 0.58; CI = 0.46–0.74; *p* ≤ 0.001) and a decrease in MIC values for erythromycin (HR = 1.30; CI = 1.10–1.54; *p* = 0.017; Table 4).

**Table 4:** Multivariable Cox proportional hazard regression model representing minimum inhibitory concentration trends of *Enterococcus faecalis* and *Enterococcus faecium* to different antimicrobials in this study, in canine clinical infections at the Cornell University Animal Health Diagnostic Center (AHDC), 2007–2020.

	*Enterococcus faecalis*					*Enterococcus faecium*				
	Hazard ratio	Probability of lower MIC^b^	*p*-value	adj. *p*-value^c^	95% CI	Hazard ratio	Probability of lower MIC^b^	*p*-value	adj. *p*-value^c^	95% CI
Ampicillin										
Urinary tract^a^										
Intestinal	1.02		0.919	0.968	0.73–1.42	1.33		0.186	0.516	0.87–2.02
Invasive	0.92		0.592	0.816	0.67–1.26	1.25		0.240	0.567	0.86–1.80
Unspecified site	1.44		0.040	0.160	1.02–2.03	1.63		0.346	0.683	0.59–4.49
Reproductive system	1.18		0.227	0.561	0.90–1.54	**3.86**	**0.79**	**<0.001**	**<0.001**	**2.26–6.58**
Skin and soft tissues	1.14		0.233	0.561	0.92–1.40	**1.74**	**0.64**	**0.003**	**0.018**	**1.21–2.51**
Isolation date (2007–2010)^a^										
Isolation date (2011–2014)	0.93		0.516	0.759	0.73–1.17	1.11		0.504	0.759	0.82–1.49
Isolation date (2015–2017)	**0.65**	**0.39**	**<0.001**	**0.001**	**0.52–0.81**	**0.48**	**0.32**	**<0.001**	**<0.001**	**0.34–0.67**
Isolation date (2018–2020)	**0.71**	**0.42**	**0.002**	**0.011**	**0.58–0.88**	**0.34**	**0.25**	**<0.001**	**<0.001**	**0.24–0.46**
Penicillin G										
Urinary tract^a^										
Intestinal	0.97		0.921	0.968	0.51–1.83	1.41		0.321	0.670	0.72–2.77
Invasive	0.82		0.538	0.775	0.44–1.54	1.16		0.663	0.862	0.59–2.27
Unspecified site	1.09		0.793	0.899	0.57–2.08	1.89		0.407	0.724	0.42–8.52
Reproductive system	1.02		0.951	0.974	0.55–1.90	**3.85**	**0.79**	**<0.001**	**0.002**	**1.87–7.95**
Skin and soft tissues	0.90		0.722	0.896	0.49–1.63	1.73		0.100	0.328	0.90–3.33
Isolation date (2007–2010)^a^										
Isolation date (2011–2014)	1.17		0.311	0.670	0.87–1.57	1.17		0.520	0.759	0.73–1.87
Isolation date (2015–2017)	0.74		0.016	0.077	0.58–0.94	**0.49**	**0.33**	**0.006**	**0.035**	**0.30–0.82**
Isolation date (2018–2020)	**0.58**	**0.37**	**<0.001**	**<0.001**	**0.46–0.74**	**0.38**	**0.27**	**<0.001**	**0.002**	**0.23–0.63**
*Gentamicin*										
Urinary tract^a^										
Intestinal	0.91		0.782	0.899	0.46–1.78	1.13		0.683	0.870	0.64–1.99
Invasive	0.78		0.484	0.758	0.38–1.58	1.10		0.772	0.899	0.59–2.06
Unspecified site	0.89		0.732	0.899	0.45–1.75	0.92		0.948	0.994	0.08–11.1
Reproductive system	0.91		0.754	0.899	0.49–1.69	0.70		0.458	0.758	0.28–1.78
Skin and soft tissues	0.71		0.260	0.604	0.40–1.28	1.18		0.599	0.816	0.64–2.16
Isolation date (2007–2010)^a^										
Isolation date (2011–2014)	**0.02**	**0.02**	**0.001**	**0.009**	**0.00–0.22**	0.23		0.043	0.167	0.05–0.95
Isolation date (2015–2017)	**0.25**	**0.20**	**<0.001**	**<0.001**	**0.20–0.32**	0.03		0.403	0.724	0.00–116
Isolation date (2018–2020)	1.04		0.758	0.899	0.82–1.32	1.31		0.186	0.516	0.88–1.96
Tetracycline										
Urinary tract^a^										
Intestinal	0.92		0.664	0.862	0.63–1.35	0.75		0.186	0.516	0.49–1.15
Invasive	1.21		0.269	0.616	0.86–1.69	0.84		0.510	0.759	0.51–1.40
Unspecified site	1.01		0.953	0.974	0.71–1.43	0.99		0.995	0.995	0.04–24.7
Reproductive system	0.96		0.763	0.899	0.71–1.28	0.54		0.654	0.862	0.04–7.95
Skin and soft tissues	1.09		0.360	0.701	0.91–1.30	0.63		0.103	0.328	0.36–1.10
Isolation date (2007–2010)^a^										
Isolation date (2011–2014)	0.92		0.405	0.724	0.77–1.11	0.96		0.812	0.907	0.68–1.35
Isolation date (2015–2017)	0.88		0.125	0.389	0.75–1.04	0.79		0.224	0.561	0.54–1.15
Isolation date (2018–2020)	0.92		0.346	0.683	0.78–1.09	1.25		0.206	0.549	0.88–1.77
Doxycycline										
Urinary tract^a^										
Intestinal	1.00		0.992	0.995	0.63–1.61	0.76		0.314	0.670	0.45–1.29
Invasive	0.82		0.423	0.734	0.51–1.32	0.83		0.476	0.758	0.50–1.39
Unspecified site	0.94		0.786	0.899	0.60–1.47	1.12		0.840	0.926	0.38–3.30
Reproductive system	0.86		0.470	0.758	0.57–1.29	1.11		0.758	0.899	0.56–2.20
Skin and soft tissues	0.99		0.950	0.974	0.67–1.45	1.16		0.522	0.759	0.73–1.84
Isolation date (2007–2010)^a^										
Isolation date (2011–2014)	0.92		0.517	0.759	0.72–1.18	1.20		0.477	0.758	0.73–1.99
Isolation date (2015–2017)	0.80		0.082	0.289	0.62–1.03	0.75		0.321	0.670	0.43–1.32
Isolation date (2018–2020)	1.06		0.717	0.896	0.78–1.44	1.64		0.211	0.552	0.76–3.54
Erythromycin										
Urinary tract^a^										
Intestinal	0.84		0.469	0.758	0.51–1.36	1.03		0.884	0.944	0.65–1.63
Invasive	0.72		0.173	0.508	0.45–1.16	0.82		0.405	0.724	0.51–1.31
Unspecified site	0.71		0.160	0.480	0.45–1.14	1.33		0.473	0.758	0.61–2.85
Reproductive system	0.57		0.018	0.085	0.36–0.91	**2.15**	**0.68**	**0.004**	**0.026**	**1.27–3.64**
Skin and soft tissues	0.69		0.090	0.310	0.45–1.06	1.44		0.093	0.312	0.94–2.20
Isolation date (2007–2010)^a^										
Isolation date (2011–2014)	0.98		0.846	0.926	0.84–1.15	1.26		0.234	0.561	0.86–1.84
Isolation date (2015–2017)	1.09		0.303	0.670	0.93–1.28	0.62		0.013	0.069	0.42–0.91
Isolation date (2018–2020)	**1.30**	**0.56**	**0.003**	**0.017**	**1.10–1.54**	**0.58**	**0.37**	**0.006**	**0.035**	**0.39–0.86**
Chloramphenicol										
Urinary tract^a^										
Intestinal	0.79		0.483	0.758	0.41–1.53	0.84		0.407	0.724	0.55–1.27
Invasive	1.15		0.678	0.870	0.60–2.20	0.77		0.201	0.546	0.51–1.15
Unspecified site	0.83		0.581	0.816	0.43–1.60	0.92		0.856	0.927	0.37–2.26
Reproductive system	0.86		0.634	0.845	0.46–1.60	1.17		0.624	0.840	0.63–2.16
Skin and soft tissues	0.95		0.877	0.942	0.52–1.75	0.81		0.336	0.682	0.54–1.24
Isolation date (2007–2010)^a^										
Isolation date (2011–2014)	0.78		0.011	0.058	0.64–0.94	1.16		0.418	0.734	0.81–1.65
Isolation date (2015–2017)	**0.69**	**0.41**	**<0.001**	**0.002**	**0.57–0.83**	0.70		0.035	0.147	0.50–0.97
Isolation date (2018–2020)	0.91		0.384	0.724	0.72–1.13	0.86		0.406	0.724	0.61–1.22
Enrofloxacin										
Urinary tract^a^										
Intestinal	0.89		0.333	0.682	0.70–1.13	**0.50**	**0.33**	**<0.001**	**0.004**	**0.34–0.74**
Invasive	0.77		0.038	0.158	0.61–0.99	0.66		0.026	0.118	0.45–0.95
Unspecified site	0.77		0.015	0.072	0.62–0.95	0.98		0.966	0.980	0.44–2.18
Reproductive system	0.80		0.028	0.122	0.66–0.98	**1.92**	**0.66**	**0.007**	**0.041**	**1.19–3.08**
Skin and soft tissues	0.91		0.081	0.289	0.82–1.01	1.12		0.456	0.758	0.83–1.51
Isolation date (2007–2010)^a^										
Isolation date (2011–2014)	0.87		0.055	0.207	0.75–1.00	0.82		0.232	0.561	0.58–1.14
Isolation date (2015–2017)	**0.75**	**0.43**	**<0.001**	**0.001**	**0.64–0.87**	**0.59**	**0.37**	**0.002**	**0.016**	**0.43–0.83**
Isolation date (2018–2020)	0.89		0.127	0.389	0.76–1.03	**0.39**	**0.28**	**<0.001**	**<0.001**	**0.28–0.53**
Rifampin										
Urinary tract^a^										
Intestinal	0.95		0.849	0.926	0.55–1.64	0.92		0.782	0.899	0.50–1.68
Invasive	1.15		0.600	0.816	0.68–1.96	0.74		0.319	0.670	0.41–1.34
Unspecified site	1.17		0.560	0.798	0.69–1.99	1.21		0.716	0.896	0.43–3.41
Reproductive system	0.83		0.490	0.759	0.50–1.39	1.82		0.078	0.287	0.94–3.54
Skin and soft tissues	1.07		0.790	0.899	0.66–1.73	1.16		0.601	0.816	0.66–2.03
Isolation date (2007–2010)^a^										
Isolation date (2011–2014)	**0.69**	**0.41**	**<0.001**	**0.002**	**0.57–0.84**	0.95		0.805	0.906	0.61–1.46
Isolation date (2015–2017)	**0.55**	**0.35**	**<0.001**	**<0.001**	**0.45–0.67**	**0.34**	**0.25**	**<0.001**	**0.001**	**0.20–0.57**
Isolation date (2018–2020)	**0.69**	**0.41**	**<0.001**	**0.002**	**0.56–0.84**	**0.40**	**0.29**	**<0.001**	**0.002**	**0.25–0.65**

a Reference group.

b Probability = HR/(1+ HR)—calculated if *p*-value<0.05 (e.g., a hazard ratio of 0.5 corresponds to a 0.33 chance of an isolate at this condition having a lower MIC value compared to an isolate in the reference group).

c *p*-values were adjusted according to Benjamini and Hochberg (1995).

Bold values are significant results (*p*<0.05 after Benjamini and Hochberg adjustment).

MLR models revealed several potential associations between drug resistances. Among all *Enterococcus* isolates, ampicillin was a strong predictor of erythromycin resistance (and vice versa; OR = 8.77; 95% CI = 3.85 to 25.3; *p* < 0.001), tetracycline (OR = 5.67; 95% CI = 2.99 to 10.8; *p* < 0.001) and enrofloxacin (OR = 21.1; 95% CI = 6.28 to 132; *p* < 0.001; Table 5). Tetracycline resistance was associated with resistance to erythromycin (OR = 2.98; 95% CI = 1.57 to 6.10; *p* = 0.004) and chloramphenicol (OR = 62.6; 95% CI = 19.5 to 282; *p* < 0.001), but was found to be associated with a decrease in the probability of resistance to enrofloxacin (OR = 0.30; 95% CI = 0.17 to 0.52; *p* < 0.001) and rifampin (OR = 0.31; 95% CI = 0.19 to 0.52; *p* < 0.001) among all *Enterococcus* spp. (and vice versa). Some species differences in associations between resistances were observed. Enrofloxacin-resistance among *E. faecalis* isolates was predicted by resistance to rifampin (OR = 2.82; 95% CI = 1.86 to 4.29; *p* < 0.001) and tetracycline (OR = 0.23; 95% CI = 0.12–0.45; *p* < 0.001) and enrofloxacin-resistant *E. faecium* was only predicted by resistance to erythromycin (OR = 26.4; 95% CI = 3.10 to 581; *p* = 0.015). Resistance to erythromycin is only associated with resistance to tetracycline among *E. faecalis* (OR = 3.35; 95% CI = 1.47 to 9.04; *p* = 0.015) and to enrofloxacin among *E. faecium* (OR = 17.5; 95% CI = 2.62 to 162; *p* = 0.010) isolates.

**Table 5:** Association between resistance to ampicillin, tetracycline, erythromycin, chloramphenicol, enrofloxacin, or rifampin and other antimicrobial compounds among *Enterococcus* spp. isolates using multivariable logistic regression models.

	*Enterococcus* spp.				*Enterococcus faecalis*				*Enterococcus faecium*			
	Model^a^				Model^a^				Model^a^			
	Adj. OR	95% CI	*p*-value	Adj. *p*-value^b^	Adj. OR	95% CI	*p*-value	Adj. *p*-value^b^	Adj. OR	95% CI	*p*-value	Adj. *p*-value^b^
Resistance to ampicillin												
Resistance to tetracycline	**5.54**	**2.90–10.6**	**<0.001**	**<0.001**								
Resistance to erythromycin	**8.61**	**3.75–24.9**	**<0.001**	**<0.001**	6*10^7^	0-Inf	0.996	0.996	8.69	1.23–173	0.057	0.083
Resistance to chloramphenicol	0.25	0.05–0.88	0.047	0.075								
Resistance to enrofloxacin	**20.4**	**6.04–128**	**<0.001**	**<0.001**								
Resistance to rifampin	1.69	0.94–3.19	0.088	0.114	5*10^7^	0-Inf	0.996	0.996	3*10^7^	0-Inf	0.991	0.996
Resistance to tetracycline												
Resistance to ampicillin	**5.67**	**2.99–10.8**	**<0.001**	**<0.001**								
Resistance to erythromycin	**2.98**	**1.57–6.10**	**0.002**	**0.004**	**3.44**	**1.49–9.36**	**0.007**	**0.015**				
Resistance to chloramphenicol	**62.6**	**19.5–282**	**<0.001**	**<0.001**	**207**	**38.7–3869**	**<0.001**	**<0.001**	10.9	1.21–234	0.049	0.075
Resistance to enrofloxacin	**0.30**	**0.17–0.52**	**<0.001**	**<0.001**	**0.23**	**0.12–0.46**	**<0.001**	**<0.001**				
Resistance to rifampin	**0.31**	**0.19–0.52**	**<0.001**	**<0.001**	**0.42**	**0.21–0.82**	**0.011**	**0.020**	**0.19**	**0.07–0.48**	**<0.001**	**0.001**
Resistance to erythromycin												
Resistance to ampicillin	**8.77**	**3.85–25.3**	**<0.001**	**<0.001**	10^7^	0-Inf	0.991	0.996	8.69	1.23–173	0.057	0.083
Resistance to tetracycline	**2.66**	**1.43–5.32**	**0.003**	**0.007**	**3.35**	**1.47–9.04**	**0.008**	**0.015**				
Resistance to chloramphenicol	4*10^6^	0-Inf	0.974	0.996	3*10^6^	0-Inf	0.978	0.996				
Resistance to enrofloxacin									**17.5**	**2.62–162**	**0.005**	**0.010**
Resistance to rifampin					0.64	0.41–0.98	0.045	0.074				
Resistance to chloramphenicol												
Resistance to ampicillin	0.24	0.05–0.84	0.040	0.067								
Resistance to tetracycline	**61.7**	**19.4–278**	**<0.001**	**<0.001**	**225**	**41.5–4212**	**<0.001**	**<0.001**	9.21	1.13–190	0.059	0.083
Resistance to erythromycin	2*10^7^	0-Inf	0.987	0.996	4*10^7^	0-Inf	0.992	0.996				
Resistance to enrofloxacin	2.23	0.80–6.78	0.137	0.165	2.88	0.93–9.52	0.072	0.097				
Resistance to rifampin												
Resistance to enrofloxacin												
Resistance to ampicillin	**21.1**	**6.28–132**	**<0.001**	**<0.001**					3*10^8^	0-Inf	0.994	0.996
Resistance to tetracycline	**0.34**	**0.21–0.56**	**<0.001**	**<0.001**	**0.23**	**0.12–0.45**	**<0.001**	**<0.001**	0.12	0.01–1.19	0.091	0.114
Resistance to erythromycin									**26.4**	**3.10–581**	**0.007**	**0.015**
Resistance to chloramphenicol					2.42	0.78–8.02	0.134	0.164				
Resistance to rifampin	**2.66**	**1.84–3.84**	**<0.001**	**<0.001**	**2.82**	**1.86–4.29**	**<0.001**	**<0.001**				
Resistance to rifampin												
Resistance to ampicillin	1.70	0.97–3.14	0.074	0.097								
Resistance to tetracycline	**0.35**	**0.22–0.56**	**<0.001**	**<0.001**	**0.47**	**0.26–0.85**	**0.012**	**0.022**	**0.21**	**0.08–0.54**	**0.001**	**0.003**
Resistance to erythromycin					0.66	0.42–1.01	0.062	0.085				
Resistance to chloramphenicol												
Resistance to enrofloxacin	**2.61**	**1.81–3.77**	**<0.001**	**<0.001**	**2.86**	**1.88–4.35**	**<0.001**	**<0.001**	MP	1.20–60.8	0.036	0.063

a The model started with ampicillin, tetracycline, erythromycin, chloramphenicol, enrofloxacin, and rifampin before backwards selection; selected antimicrobials regularly tested on *Enterococcus* spp. isolates were entered in the model as explanatory variables.

b *p*-values were adjusted according to Benjamini and Hochberg (1995). Values >10^100^ and <0.01 are mentioned as Inf and 0, respectively.

Bold values are significant results (*p*<0.05 after Benjamini and Hochberg adjustment).

## 4. Discussion

The present study provided updated data on the most frequently isolated *Enterococcus* spp. from canine infections and their associated AMR patterns and trends in the northeastern United States. Antimicrobial-resistant enterococcal infections have become a major public health concern to modern health care, representing a growing global threat to human and animal health (Ahmed and Baptiste, 2018; Wada et al., 2021; Murray et al., 2022). *Enterococcus faecalis* was the most prevalent species (67.1%) encountered in dog enterococcal infections, followed by *E. faecium* (20.4%). This distribution is consistent with previous data showing that *E. faecalis* was the most commonly cultured enterococcal species (38%–77.4%) from dogs followed by *E. faecium* (12.9%–21%) in the United States (Jackson et al., 2009; KuKanich and Lubbers, 2015), as well as in other countries such as Spain (90.2 and 7.8%, respectively; Li et al., 2021) and Portugal (95.8% and 4.2%, respectively; Oliveira et al., 2016). However, an older study at the Michigan State University Veterinary Teaching Hospital performed between 1996 and 1998, including a low number of canine enterococcal isolates (*N* = 35), described a predominance of *E. faecium* (37.1%), followed by *E. gallinarum* (31.4%) and *E. faecalis* (20%; Simjee et al., 2002). *E. gallinarum*, which is a dominant bacterium in poultry gastrointestinal tracts, was rarely found in our study (0.8%). Similarly, *E. casseliflavus* was rarely reported (1%), and *E. flavescens* was not found. In contrast to previous data from dogs in Athens, Georgia, United States (Jackson et al., 2009) and Eastern Slovakia (Kubašová et al., 2017), the zoonotic pathogen commonly found in animals, *E. hirae*, was rarely observed (0.8%) in this study.

Due to limited therapeutic options, enterococci are hard to treat with antimicrobial agents, even when relatively susceptible isolates are involved. Although uncomplicated urinary infections are easily treated empirically with a first-line antimicrobial, typically a penicillin, cephalosporin, or folate-pathway antagonist (Weese et al., 2019), *Enterococcus* spp. possess inherent resistance to cephalosporins (e.g., cephalexin, cefazolin, cefovecin, cefpodoxime, ceftiofur) through the expression of low-affinity penicillin binding proteins (PBP4 in *E. faecalis* and PBP5 in *E. faecium*) that bind weakly to these antimicrobials (Hollenbeck and Rice, 2012). *Enterococcus faecium* isolates also possess an inherent resistance to penicillins and carbapenems. The activity of fluoroquinolones (e.g., enrofloxacin, pradofloxacin, orbifloxacin, marbofloxacin) in urine against enterococci is controversial, and the International Society for Companion Animal Infectious Diseases (ISCAID) recommended to avoid these drugs in the management of enterococcal urinary infections in dogs (de Lastours et al., 2017; Weese et al., 2019). Acquired resistance can also occur in enterococci through sporadic mutations or the acquisition of mobile genetic elements, complicating treatment of enterococcal infections (Oliveira et al., 2020).

Overall, alarming proportions of canine clinical enterococcal isolates were MDR. Compared to our findings, data from South Africa showed higher resistance rates to ampicillin (41.2%), penicillin (45.5%), and chloramphenicol (26.3%), but lower resistance rates against enrofloxacin among enterococci (58%; Oguttu et al., 2021). A Spanish study also revealed a lower percentage of resistance to enrofloxacin (~30%) and higher rates of resistance to chloramphenicol (~13%; Li et al., 2021). However, a recent Polish study described higher resistance rates to all the tested antimicrobials, with 92.2% to enrofloxacin, 90.2% to erythromycin, 88.2% to tetracycline, and 56.9% to chloramphenicol (Stępień-Pyśniak et al., 2021). Resistance rates among enterococci are directly related to the distribution of species (Hollenbeck and Rice, 2012; Mercuro et al., 2018; Zhou et al., 2020). *Enterococcus faecium* is recognized to have a higher prevalence of resistance to multiple antimicrobials of both clinical and veterinary significance, particularly beta-lactams, tetracycline, fluoroquinolones, and nitrofurantoin, while *E. faecalis* isolates are more likely to express virulence genes but retain a relatively lower prevalence of resistance to antimicrobials (Johnston and Jaykus, 2004; Zaheer et al., 2020); thus, the resistance rates among enterococci are typically higher in studies in which *E. faecium* has a relatively high prevalence rate. Interestingly, compared to previous studies performed in the United States (Simjee et al., 2002; Jackson et al., 2009; KuKanich and Lubbers, 2015), this study showed that *E. faecalis* isolates have higher resistance rates to enrofloxacin, erythromycin, and tetracycline. Regarding *E. faecium*, isolates from the previous studies showed similar percentage of resistance to penicillins, but higher resistance rates to enrofloxacin, erythromycin, tetracycline, and nitrofurantoin (Simjee et al., 2002; Jackson et al., 2009; KuKanich and Lubbers, 2015). The widespread resistance of enterococci to antimicrobials has without a doubt a substantial impact on the empirical and definitive antimicrobial use and spread of MDR bacteria in the United States.

Vancomycin-resistant enterococci (VRE) have become among the priority pathogens reported by the World Health Organization (Cassini et al., 2019). The CDC categorized VRE as serious threats to current healthcare practices, suggesting the need for increased monitoring and prevention activities (Weiner et al., 2016). Unlike recent data from the United States that described a shocking prevalence of vancomycin-resistant *E. faecium* nosocomial isolates in human medicine, ranging between 75 and 80% (Zhou et al., 2020), our findings showed a low percentage of vancomycin resistance among *E. faecium* isolates (0.8%), as well as *E. faecalis* isolates (0.3%). Higher proportion of vancomycin resistance (54%) was observed among other species, which can be explained by the predominance of *E. gallinarum* group isolates accounting for 67 out of 68 VRE other than *E. faecalis* and *E. faecium* (Table 1). The gallinarum group consisting of the species *E. gallinarum*, *E. casseliflavus*, and *E. flavescens*, possesses intrinsic low-level resistance to vancomycin by synthesis of modified peptidoglycan precursors ending in D-alanine-D-serine (*via* the *vanC* gene), but they are responsible only for a minor percentage of enterococcal infections (Monticelli et al., 2018). Overall, five enterococcal isolates with acquired vancomycin resistance were found, belonging to *E. faecalis* (*N* = 2)*, E. faecium* (*N* = 2), and *E. canintestini* (*N* = 1) and mainly occurred in skin and soft tissues infections (Supplementary Table S2). All the vancomycin-resistant isolates had MDR patterns, and one was XDR. VRE infections, especially MDR and XDR strains, have become a global public health challenge in human and veterinary medicine involving both drug kinetics and bacterial resistance factors; these infections are often difficult-to-treat and may sometimes be life threatening because there are fewer antimicrobials that can fight these resistant bacteria (Patel and Gallagher, 2015; Zhou et al., 2020). Since vancomycin was not tested until 2017 in the Cornell University AHDC, the presence of VRE in the canine population is probably underestimated. However, interestingly, four out the five vancomycin-resistant isolates were isolated in 2020. To date, VRE remain rare in animals; thus, the recent detection of four resistant isolates in the same year represents an early warning sign on the dissemination of this serious threat between dogs and their environment (Jackson et al., 2009; KuKanich and Lubbers, 2015; Amachawadi et al., 2018; Dungan and Bjorneberg, 2021; Jeamsripong et al., 2021).

Although our MKT, Sen’s slope, MLR, and/or survival analysis models suggested that resistance to multiple antimicrobials such as penicillins, enrofloxacin, and rifampin in enterococci is increasing, resistance to tetracyclines is on a downward trajectory compared to AMR data from the last decade. The decrease in the frequency of use of tetracycline may be associated with the decrease in resistance to this antimicrobial class. Unlike penicillins and fluoroquinolones, which are the most commonly prescribed antimicrobial drug classes, tetracyclines are rarely prescribed at Cornell University Hospital for Animals emergency (6%) and critical care (0.8%) services (Robbins et al., 2020). These findings are similar to those recently reported in primary care and specialty practice across three academic veterinary hospitals (Cornell University, North Carolina State University, and Texas A&M University) in the United States (Goggs et al., 2021). Tetracyclines are not excreted in urine at high levels in the canine population and are therefore not recommended to treat urinary infections (Weese et al., 2019). Of note, current guidelines recommend the use of tetracyclines for the treatment of mild to moderate respiratory infections and fluoroquinolones for severe cases (Lappin et al., 2017).

Genetic co-resistance could play a crucial role in selecting resistant bacteria and promoting AMR. For example, our MLR model revealed that tetracycline is significantly associated with resistance to ampicillin, erythromycin, and chloramphenicol (Table 5). Tetracycline resistance is commonly associated with the presence of plasmid-borne *tet* genes, which confer ribosomal protection or efflux pumps. Moreover, erythromycin resistance is commonly mediated by the acquisition of *erm (B)* gene located mostly on plasmids, which encodes the ribosomal RNA methylase. All these genetic determinants can be located on the same mobile genetic element (Morroni et al., 2018; Cho et al., 2020), allowing the dissemination of resistance between bacteria in ecosystems (Hollenbeck and Rice, 2012). Dogs are in close contact with their environment; thus, the transmission of drug-resistant enterococci and AMR determinants can easily occur in either direction through direct or indirect contact (Rees et al., 2021). Taken together, we suggest that establishing better hygiene in communities and enhancing the prudent use of antimicrobials, particularly ampicillin, erythromycin and tetracycline, are essential to conserve their therapeutic effects and prevent the co-selection of resistance to other antimicrobials, and consequently tackle the burden of AMR in both human and veterinary settings (Devi, 2020; Charani et al., 2021).

## 5. Limitations of the study

Our dataset did not provide individual animal identifications, only sample submission identification. Although we included only one isolate per sample submission, there could be more than one isolate per patient in the analyzed data. It is important to note that interpretative criteria, specific to dogs, for resistance in enterococci are not available. Only human interpretive criteria are available from CLSI (CLSI, 2020). There is a dog-specific breakpoint for enrofloxacin and streptococci (both the veterinary antibiogram committee of the French Society for Microbiology (CA-SFM, 2021) and CLSI VAST provide the same breakpoint (CLSI, 2020)). We applied this streptococci breakpoint to the enterococci isolates. The antimicrobials interpreted with human breakpoints may not reflect the true prevalence of clinical resistance in dogs because of differences in human and canine antimicrobial pharmacokinetics. Enrofloxacin resistance may be underestimated or overestimated if the enterococci and streptococci have significantly different enrofloxacin pharmacodynamics. However, we expect trends within each antimicrobial to be reliable. Due to the retrospective design of this investigation, we were unable to assess the susceptibility of antimicrobials in all body sites, test other antimicrobials (particularly teicoplanin, daptomycin, linezolid, tedizolid, quinupristin/dalfopristin, fosfomycin, tigecycline, and eravacyline), or to collect more sociodemographic, behavioral, and clinical data that could be associated with resistance patterns. We have lower confidence in the trends of the less-prevalent *Enterococcus* species (i.e., not *Enterococcus faecalis* and not *Enterococcus faecium*) due to the smaller number of isolates. Vancomycin resistance was underestimated since this antibiotic was not tested until 2017 and was not tested in all isolates after that period. Furthermore, we were unable to perform additional phenotypic (e.g., nitrocefin test) and molecular (e.g., whole genome sequencing) analysis to confirm the initial species identification, determine the AMR determinants, and identify the *Enterococcus* clones circulating in the northeastern United States. Molecular typing is critical to better understand the current epidemiology of *Enterococcus* in humans and animals from a One Health approach and, therefore, to preserve the effectiveness of existing antimicrobials and reinforce antimicrobial stewardship interventions.

## 6. Conclusion

We provided a relevant update and an epidemiological evidence base for enterococci AMR patterns for veterinarians in the northeastern United States. Antimicrobial resistant canine enterococci, particularly vancomycin-resistant isolates, are a major public health threat to both human and veterinary medicine. Hence, the critical need to make rational prescribing decisions on the prudent use of antimicrobials and improve the quality of care for patients, especially when empirical antimicrobial treatment for enterococcal infection is common. To better understand the local epidemiology of drug-resistant enterococci and ensure effective treatment, further studies including a large number of human, animal, and environmental samples and aiming to assess other antimicrobials of clinical and veterinary interest, investigate the genetic determinants of AMR, identify the circulating clones, and suggest antimicrobial stewardship interventions are required.

## Data availability statement

The datasets presented in this study can be found in online repositories. The names of the repository/repositories and accession number(s) can be found at: 10.5281/zenodo.7126369.

## Author contributions

MO: conceptualization, methodology, software, formal analysis, validation, data curation, visualization, writing—original draft, and writing—review and editing. CA: investigation, resources, data curation, and writing—review and editing. CC: conceptualization, methodology, software, validation, resources, data curation, supervision, administration, and writing—review and editing. All authors contributed to the article and approved the submitted version.

## Funding

MO is supported by the Atkinson Postdoctoral Fellowship (Cornell University).

## Conflict of interest

The authors declare that the research was conducted in the absence of any commercial or financial relationships that could be construed as a potential conflict of interest.

## Publisher’s note

All claims expressed in this article are solely those of the authors and do not necessarily represent those of their affiliated organizations, or those of the publisher, the editors and the reviewers. Any product that may be evaluated in this article, or claim that may be made by its manufacturer, is not guaranteed or endorsed by the publisher.

## Supplementary material

The Supplementary material for this article can be found online at: https://www.frontiersin.org/articles/10.3389/fmicb.2022.1025242/full#supplementary-material
