# Supplementary material for: Antimicrobial resistance among canine enterococci in the northeastern United States, 2007–2020
Source: Front Microbiol. 2023 Jan 5;13:1025242. doi: 10.3389/fmicb.2022.1025242 (PMC9849698; doi:10.3389/fmicb.2022.1025242)
Supplement: Supplementary file 1 [file Table_1.DOCX]

**Supplementary Table S1.** Multivariable Cox proportional hazard regression model representing minimum inhibitory concentration trends of *Enterococcus* spp. to different antimicrobials in this study, in canine clinical infections at the Cornell University Animal Health Diagnostic Center (AHDC), 2007-2020.

|  | **Hazard ratio** | **Probability of lower MIC^¶^** | **P-value** | **adj. P-value^§^** | **95% CI** |
| --- | --- | --- | --- | --- | --- |
| ***Ampicillin (N = 3589 isolates)*** | | | | | |
| ***Enterococcus faecalis*^1^** |  |  |  |  |  |
| ***Enterococcus faecium*** | **0.14** | **0.12** | **<0.001** | **<0.001** | **0.12-0.17** |
| ***Enterococcus* spp.** | 0.99 |  | 0.923 | 0.952 | 0.75-1.20 |
| **Urinary tract^1^** |  |  |  |  |  |
| **Intestinal** | 0.89 |  | 0.173 | 0.347 | 0.75-1.05 |
| **Invasive** | 0.85 |  | 0.142 | 0.318 | 0.69-1.05 |
| **Unspecified site** | 1.13 |  | 0.252 | 0.428 | 0.92-1.38 |
| **Reproductive system** | 1.23 |  | 0.033 | 0.104 | 1.02-1.48 |
| **Skin and soft tissues** | 1.11 |  | 0.119 | 0.289 | 0.97-1.26 |
| **Isolation date (2007-2010)^1^** |  |  |  |  |  |
| **Isolation date (2011-2014)** | 1.10 |  | 0.239 | 0.428 | 0.94-1.28 |
| **Isolation date (2015-2017)** | **0.79** | **0.44** | **0.001** | **0.005** | **0.68-0.91** |
| **Isolation date (2018-2020)** | **0.77** | **0.44** | **<0.001** | **0.001** | **0.67-0.88** |
| ***Penicillin G (N = 2327 isolates)*** | | | | | |
| ***Enterococcus faecalis*^1^** |  |  |  |  |  |
| ***Enterococcus faecium*** | **0.13** | **0.12** | **<0.001** | **<0.001** | **0.11-0.16** |
| ***Enterococcus* spp.** | **1.64** | **0.62** | **0.001** | **0.004** | **1.22-2.19** |
| **Urinary tract^1^** |  |  |  |  |  |
| **Intestinal** | 1.04 |  | 0.854 | 0.894 | 0.69-1.56 |
| **Invasive** | 0.86 |  | 0.515 | 0.649 | 0.56-1.34 |
| **Unspecified site** | 1.06 |  | 0.822 | 0.883 | 0.66-1.70 |
| **Reproductive system** | 1.28 |  | 0.263 | 0.430 | 0.83-1.97 |
| **Skin and soft tissues** | 1.16 |  | 0.455 | 0.593 | 0.78-1.73 |
| **Isolation date (2007-2010)^1^** |  |  |  |  |  |
| **Isolation date (2011-2014)** | 1.21 |  | 0.073 | 0.206 | 0.98-1.48 |
| **Isolation date (2015-2017)** | 0.87 |  | 0.122 | 0.289 | 0.73-1.04 |
| **Isolation date (2018-2020)** | **0.71** | **0.42** | **<0.001** | **<0.001** | **0.60-0.84** |
| ***Gentamicin (N = 1913 isolates)*** | | | | | |
| ***Enterococcus faecalis*^1^** |  |  |  |  |  |
| ***Enterococcus faecium*** | 1.06 |  | 0.606 | 0.718 | 0.84-1.34 |
| ***Enterococcus* spp.** | **4.73** | **0.83** | **<0.001** | **<0.001** | **3.17-7.05** |
| **Urinary tract^1^** |  |  |  |  |  |
| **Intestinal** | 0.91 |  | 0.703 | 0.801 | 0.56-1.48 |
| **Invasive** | 0.74 |  | 0.257 | 0.428 | 0.44-1.24 |
| **Unspecified site** | 0.87 |  | 0.595 | 0.714 | 0.52-1.45 |
| **Reproductive system** | 0.90 |  | 0.678 | 0.792 | 0.55-1.47 |
| **Skin and soft tissues** | 0.83 |  | 0.406 | 0.537 | 0.53-1.29 |
| **Isolation date (2007-2010)^1^** |  |  |  |  |  |
| **Isolation date (2011-2014)** | **0.08** | **0.07** | **<0.001** | **<0.001** | **0.05-0.14** |
| **Isolation date (2015-2017)** | **0.26** | **0.21** | **<0.001** | **<0.001** | **0.20-0.33** |
| **Isolation date (2018-2020)** | 1.26 |  | 0.016 | 0.054 | 1.04-1.52 |
| ***Tetracycline (N = 2432 isolates)*** | | | | | |
| ***Enterococcus faecalis*^1^** |  |  |  |  |  |
| ***Enterococcus faecium*** | **0.46** | **0.31** | **<0.001** | **<0.001** | **0.39-0.52** |
| ***Enterococcus* spp.** | 0.88 |  | 0.127 | 0.293 | 0.74-1.04 |
| **Urinary tract^1^** |  |  |  |  |  |
| **Intestinal** | 0.92 |  | 0.580 | 0.705 | 0.70-1.22 |
| **Invasive** | 1.17 |  | 0.249 | 0.428 | 0.90-1.52 |
| **Unspecified site** | 0.99 |  | 0.931 | 0.952 | 0.73-1.34 |
| **Reproductive system** | 1.03 |  | 0.789 | 0.877 | 0.81-1.32 |
| **Skin and soft tissues** | 1.07 |  | 0.391 | 0.531 | 0.92-1.24 |
| **Isolation date (2007-2010)^1^** |  |  |  |  |  |
| **Isolation date (2011-2014)** | 0.98 |  | 0.831 | 0.883 | 0.85-1.14 |
| **Isolation date (2015-2017)** | 0.91 |  | 0.188 | 0.361 | 0.80-1.04 |
| **Isolation date (2018-2020)** | 0.98 |  | 0.731 | 0.823 | 0.85-1.13 |
| ***Doxycycline (N = 2091 isolates)*** | | | | | |
| ***Enterococcus faecalis*^1^** |  |  |  |  |  |
| ***Enterococcus faecium*** | **0.50** | **0.33** | **<0.001** | **<0.001** | **0.42-0.59** |
| ***Enterococcus* spp.** | 1.17 |  | 0.063 | 0.183 | 0.99-1.38 |
| **Urinary tract^1^** |  |  |  |  |  |
| **Intestinal** | 0.85 |  | 0.337 | 0.497 | 0.61-1.18 |
| **Invasive** | 0.76 |  | 0.086 | 0.234 | 0.55-1.04 |
| **Unspecified site** | 0.86 |  | 0.372 | 0.523 | 0.61-1.21 |
| **Reproductive system** | 0.88 |  | 0.395 | 0.531 | 0.65-1.19 |
| **Skin and soft tissues** | 0.96 |  | 0.799 | 0.877 | 0.73-1.27 |
| **Isolation date (2007-2010)^1^** |  |  |  |  |  |
| **Isolation date (2011-2014)** | 1.04 |  | 0.701 | 0.801 | 0.84-1.29 |
| **Isolation date (2015-2017)** | 0.86 |  | 0.167 | 0.342 | 0.69-1.07 |
| **Isolation date (2018-2020)** | 1.24 |  | 0.119 | 0.289 | 0.95-1.62 |
| ***Erythromycin (N = 2367 isolates)*** | | | | | |
| ***Enterococcus faecalis*^1^** |  |  |  |  |  |
| ***Enterococcus faecium*** | **0.43** | **0.30** | **<0.001** | **<0.001** | **0.37-0.50** |
| ***Enterococcus* spp.** | **1.36** | **0.58** | **0.001** | **0.002** | **1.14-1.61** |
| **Urinary tract^1^** |  |  |  |  |  |
| **Intestinal** | 0.83 |  | 0.186 | 0.361 | 0.62-1.10 |
| **Invasive** | 0.81 |  | 0.158 | 0.335 | 0.60-1.09 |
| **Unspecified site** | 0.90 |  | 0.520 | 0.649 | 0.65-1.25 |
| **Reproductive system** | 0.83 |  | 0.217 | 0.399 | 0.62-1.12 |
| **Skin and soft tissues** | 0.92 |  | 0.558 | 0.688 | 0.71-1.20 |
| **Isolation date (2007-2010)^1^** |  |  |  |  |  |
| **Isolation date (2011-2014)** | 1.07 |  | 0.368 | 0.523 | 0.92-1.24 |
| **Isolation date (2015-2017)** | 1.00 |  | 0.977 | 0.987 | 0.86-1.17 |
| **Isolation date (2018-2020)** | 1.16 |  | 0.040 | 0.121 | 1.01-1.35 |
| ***Chloramphenicol (N = 2375 isolates)*** | | | | | |
| ***Enterococcus faecalis*^1^** |  |  |  |  |  |
| ***Enterococcus faecium*** | 1.10 |  | 0.160 | 0.335 | 0.96-1.27 |
| ***Enterococcus* spp.** | **1.52** | **0.60** | **<0.001** | **<0.001** | **1.27-1.83** |
| **Urinary tract^1^** |  |  |  |  |  |
| **Intestinal** | 0.75 |  | 0.107 | 0.275 | 0.53-1.06 |
| **Invasive** | 0.84 |  | 0.294 | 0.456 | 0.60-1.16 |
| **Unspecified site** | 0.81 |  | 0.279 | 0.445 | 0.55-1.19 |
| **Reproductive system** | 0.82 |  | 0.249 | 0.428 | 0.58-1.15 |
| **Skin and soft tissues** | 0.87 |  | 0.385 | 0.531 | 0.63-1.19 |
| **Isolation date (2007-2010)^1^** |  |  |  |  |  |
| **Isolation date (2011-2014)** | 0.89 |  | 0.152 | 0.334 | 0.77-1.04 |
| **Isolation date (2015-2017)** | **0.72** | **0.42** | **<0.001** | **<0.001** | **0.62-0.84** |
| **Isolation date (2018-2020)** | 0.92 |  | 0.324 | 0.486 | 0.77-1.09 |
| ***Enrofloxacin (N = 3571 isolates)*** | | | | | |
| ***Enterococcus faecalis*^1^** |  |  |  |  |  |
| ***Enterococcus faecium*** | **0.12** | **0.11** | **<0.001** | **<0.001** | **0.10-0.14** |
| ***Enterococcus* spp.** | **0.63** | **0.39** | **<0.001** | **<0.001** | **0.55-0.72** |
| **Urinary tract^1^** |  |  |  |  |  |
| **Intestinal** | **0.68** | **0.41** | **<0.001** | **<0.001** | **0.57-0.82** |
| **Invasive** | **0.68** | **0.40** | **<0.001** | **<0.001** | **0.56-0.82** |
| **Unspecified site** | 0.79 |  | 0.018 | 0.058 | 0.64-0.96 |
| **Reproductive system** | 0.92 |  | 0.300 | 0.458 | 0.77-1.08 |
| **Skin and soft tissues** | 0.93 |  | 0.106 | 0.275 | 0.84-1.02 |
| **Isolation date (2007-2010)^1^** |  |  |  |  |  |
| **Isolation date (2011-2014)** | 0.92 |  | 0.204 | 0.382 | 0.81-1.05 |
| **Isolation date (2015-2017)** | **0.79** | **0.44** | **<0.001** | **0.001** | **0.70-0.90** |
| **Isolation date (2018-2020)** | **0.85** | **0.46** | **0.012** | **0.043** | **0.74-0.96** |
| ***Rifampin (N = 2325 isolates)*** | | | | | |
| ***Enterococcus faecalis*^1^** |  |  |  |  |  |
| ***Enterococcus faecium*** | **0.65** | **0.39** | **<0.001** | **<0.001** | **0.53-0.79** |
| ***Enterococcus* spp.** | **2.71** | **0.73** | **<0.001** | **<0.001** | **2.26-3.26** |
| **Urinary tract^1^** |  |  |  |  |  |
| **Intestinal** | 0.82 |  | 0.282 | 0.445 | 0.58-1.17 |
| **Invasive** | 0.85 |  | 0.350 | 0.508 | 0.60-1.20 |
| **Unspecified site** | 1.04 |  | 0.834 | 0.883 | 0.71-1.52 |
| **Reproductive system** | 0.89 |  | 0.511 | 0.649 | 0.63-1.26 |
| **Skin and soft tissues** | 1.00 |  | 0.987 | 0.987 | 0.74-1.36 |
| **Isolation date (2007-2010)^1^** |  |  |  |  |  |
| **Isolation date (2011-2014)** | **0.79** | **0.44** | **0.011** | **0.039** | **0.66-0.95** |
| **Isolation date (2015-2017)** | **0.61** | **0.38** | **<0.001** | **<0.001** | **0.51-0.73** |
| **Isolation date (2018-2020)** | **0.69** | **0.41** | **<0.001** | **<0.001** | **0.58-0.81** |

^1^Reference group; ^¶^Probability = HR/(1+ HR) - calculated if P-value<0.05 (e.g., a hazard ratio of 0.5 corresponds to a 0.33 chance of an isolate at this condition having a lower MIC value compared to an isolate in the reference group). ^‡^Isolates were only tested between 2015 and 2020. ^§^P-values were adjusted according to (37).

37. Benjamini Y, Hochberg Y. Controlling the False Discovery Rate: A Practical and Powerful Approach to Multiple Testing. J. R. Stat. Soc. 1995;57(1):289-300.
