# Supplementary material for: Antimicrobial resistance among canine enterococci in the northeastern United States, 2007–2020
Source: Front Microbiol. 2023 Jan 5;13:1025242. doi: 10.3389/fmicb.2022.1025242 (PMC9849698; doi:10.3389/fmicb.2022.1025242)
Supplement: Supplementary file 2 [file Table_2.DOCX]

**Supplementary Table S2.** Minimal inhibitory concentrations (MIC, mg/L) values and resistance patterns in enterococcal isolates with acquired vancomycin resistance isolated from canine clinical infections at the Cornell University Animal Health Diagnostic Center (AHDC), 2007-2020.

| **Species** | **Body Site** | **Isolation year** | **MIC (AMR pattern)** | | | | | | | | | | **AMR Phenotype** |
| --- | --- | --- | --- | --- | --- | --- | --- | --- | --- | --- | --- | --- | --- |
|  |  |  | **AMP** | **PG** | **VAN** | **TET** | **DOX** | **ERY** | **CHL** | **EFX** | **RIF** | **FUR** |  |
| ***E. faecium*** | Skin and soft tissues | 2017 | **>8 (R)** | **>8 (R)** | **>16 (R)** | >1 (ND) | >0.5 (ND) | **>4 (R)** | **>32 (R)** | **>4 (R)** | **>2 (R)** | **>64 (R)** | XDR |
| ***E. faecium*** | Skin and soft tissues | 2020 | 1 (S) | 8 (S) | **8 (R)** | ≤0.25 (S) | ≤0.12 (S) | **2 (R)** | ≤8 (S) | **2 (R)** | **2 (R)** | **64 (R)** | MDR |
| ***E. faecalis*** | Skin and soft tissues | 2020 | 1 (S) | 8 (S) | **>16 (R)** | 0.5 (S) | 0.5 (S) | **>4 (R)** | ≤8 (S) | 0.5 (S) | **2 (R)** | ≤16 (S) | MDR |
| ***E. canintestini*** | Reproductive system | 2020 | 1 (S) | 2 (S) | **8 (R)** | ≤0.25 (S) | >0.5 (ND) | **>4 (R)** | **16 (R)** | **2 (R)** | ≤1 (S) | ≤16 (S) | MDR |
| ***E. faecalis*** | Urinary tract | 2020 | 1 (S) | 4 (S) | **8 (R)** | 0.5 (S) | 0.25 (S) | 0.5 (S) | ≤8 (S) | **1 (R)** | **>2 (R)** | ≤16 (S) | MDR |

*AMP, ampicillin; PG, penicillin G; VAN, vancomycin; TET, tetracycline; DOX, doxycycline; ERY, erythromycin; CHL, chloramphenicol; EFX, enrofloxacin; RIF, rifampin; FUR, nitrofurantoin; AMR, antimicrobial resistance; MDR, multidrug-resistant; XDR, extremely-drug resistant.
